# Characterization of Ligand-Receptor Pair in Bladder Cancer Develops a Validated Scoring Model for Prognosis and Treatment Response

**DOI:** 10.3389/fcell.2022.915798

**Published:** 2022-06-17

**Authors:** Chuang Wang, Honglei Wan, Han Zhang, Bo Yang, Wen-Kuan Huang, Wenguo Sun

**Affiliations:** ^1^ Department of Urology, Guilin People’s Hospital, Guilin, China; ^2^ Department of Urology, Handan First Hospital, Handan, China; ^3^ Division of Hematology-Oncology, Department of Internal Medicine, Chang Gung Memorial Hospital at Linkou, Chang Gung University College of Medicine, Taoyuan, Taiwan; ^4^ Department of Urology, The Affiliated Hospital of Guilin Medical University, Guilin, China; ^5^ Department of Urology, The Second Affiliated Hospital of Chongqing Medical University, Chongqing, China

**Keywords:** ligand-receptor pair, bladder cancer, response prediction, gene signature, prognosis

## Abstract

The role of ligand-receptor (LR) pairs in disease progression has been explored in bladder cancer. However, the relationship of LR pairs with cancer prognosis and treatment response remains poorly understood. We characterized the LR pair network and identified three distinct molecular subtypes with distinct biologic features based on the TCGA database (*n* = 406) and validated in GSE13507 (*n* = 165) and GSE32894 (*n* = 224). Three subtypes were compared for differences in patient clinical characteristics, genomic, and transcriptomic features. A multivariate Lasso Cox regression model was applied to construct an LR pairs-based scoring model to stratify the prognostic risk of patients. We demonstrated the high LR. score patients had better responses in chemotherapy, while low LR. score patients may benefit from immune checkpoint blockade (ICB). Collectively, we identified three LR pair-related subtypes associated with prognosis. We constructed and validated a LR pairs-based gene signature, which helps to predict prognosis and differentiate the susceptible population to chemotherapy and immunotherapy in patients with bladder cancer. Among the LR pairs significantly related to prognosis, ANAX1−EGFR axis was found to be potential therapeutic target for treatment of bladder cancer.

## Introduction

Bladder cancer is the second most common urological malignancies worldwide, with 90% cases of urothelial carcinoma (Antoni et al., 2017; Loriot et al., 2019). Bladder cancer is classified as non-muscle-invasive bladder cancer (NMIBC) and muscle-invasive bladder cancer (MIBC) for distinct prognosis and treatment approaches. MIBC is featured by an aggressive state and rapid progression, which leads to high mortality (5-year survival of 10–15% in metastatic disease). The histology type grade and combined with the TNM system, are used to predict the prognosis of MIBC.

Recent advances in genomic and transcriptomic profiling revealed that MIBC is a heterogeneous disease group with distinct molecular types (Robertson et al., 2018). Various molecular subtype classifications have been developed with shared characteristics, including basal and luminal features but also different granularity of molecular traits (Blaveri et al., 2005; Choi et al., 2014; Sjodahl et al., 2017). Recently, a consensus classification including six subtypes (luminal-papillary, basal/squamous, luminal-nonspecified, luminal unstable, stroma-rich, and neuroendocrine-like) was identified by network-based analyses (Kamoun et al., 2020). While the consensus molecular classification showed the prognostic associations, the somewhat overlapping survival curves among these subtypes remained a major obstacle for clinical application.

Interaction of ligand-receptor pairs mediating cell-cell communication in the tumor microenvironment is critical for cancer cell development (Mcgranahan and Swanton, 2017). The majority of previous research focused on a single ligand-receptor (LR) pair between two cells to explore the biological effect on tumor growth (Sun et al., 2021). However, the comprehensive analysis of different cell interactions through multiple ligand-receptor pairs in bladder cancer has not been fully elucidated. Here, we illustrated the LR pair network and identified the LR pair-related gene signature associated with the prognosis of bladder cancer. We identified three distinct subtypes and characterized their clinical and biologic features. Furthermore, we constructed an LR pair-based scoring system, which demonstrated a significant ability to predict the immune checkpoint blockade (ICB) response and chemotherapy response.

## Materials and Methods

### Data Source and Pre-Processing

**FIGURE 1:**
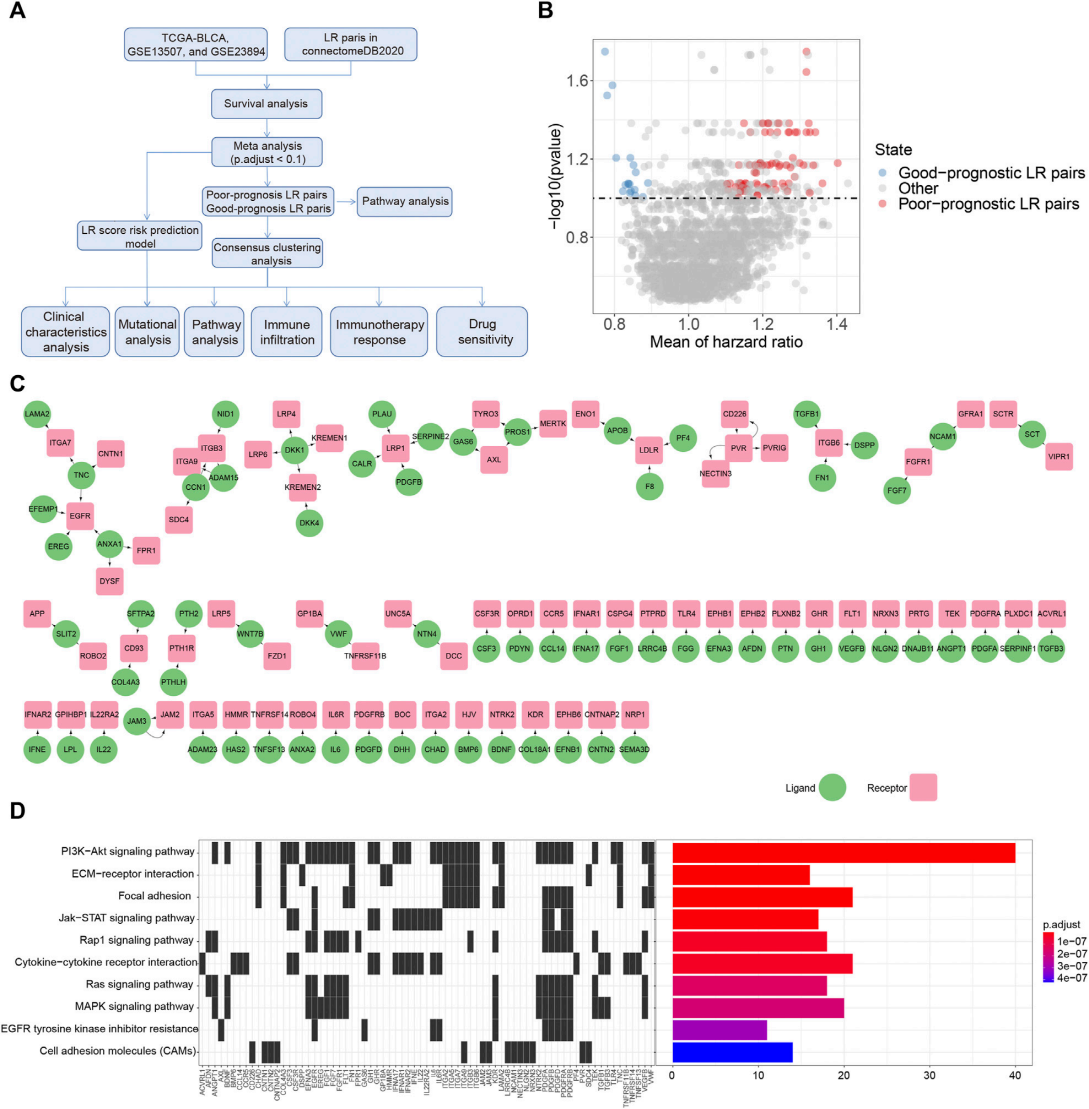
Prognostic associated LR pairs selection **(A)**. The study workflow **(B)**. Volcano plots of the distributions of the prognostic significant LR pairs. Red and blue color represent good- and poor-prognostic LR genes, respectively **(C)** Interaction network plot of prognostic significant LR pairs. Red and green color represent receptors and ligands, respectively **(D)**. Top 10 KEGG pathway enrichment results of prognostic significant LR pairs.

We downloaded clinical and gene expression data of the TCGA dataset for bladder cancer as a training cohort from UCSC Xena (https://xenabrowser.net). The gene expression profile of each tumor was extracted separately and mapped to the genome annotation file (hg38). We investigated the transcriptional data in Transcripts Per Kilobase Million (TPM) values, and log2 [(TPM) + 1] transformations were performed on gene expression units for downstream analyses. Samples without gene expression data or clinical information were excluded, leading to a final sample size of 406 patients. Regarding the validation cohorts, two published datasets with gene expression data and clinical information were obtained, including GSE13507 (Kim et al., 2010; Lee et al., 2010) and GSE32894 (Sjodahl et al., 2012). We extracted the primary bladder cancer samples with clinical data and normalized gene expression profiles, and removed the probes with no gene detection values, resulting in 165 and 224 samples, respectively. Additionally, a total of 2293 Ligand Receptor (LR) pairs were acquired from the connectomeDB2020 database (Hou et al., 2020) (Supplementary Table S1). The workflow of this study is shown in Figure 1A.

### Patients Stratification and Survival Analysis

For each LR pair, a patient was defined as “high” if the sum of the gene expression of the LR pairs was equal to or greater than the median of the sum of the gene expression of all patient LR pairs. Otherwise, the patient was assigned as “low.” The expression of LR was calculated based on the sum of the expression of two genes in a pair. The overall survival probabilities of patients were analyzed using the “Survival” package in R (version 4.0.3). Statistical significance was assessed by a log-rank test. Hazard ratios (HR) were calculated by indexing coefficients from Cox regression models. We performed a survival analysis for each cohort. We combined the *p*-values of the three cohorts by the “Edgington” method using the “sump” function in the “metap” package (version 1.4) for the meta-analysis. Lastly, “Storey’s" method (Storey, 2002) was used for multiple testing corrections using the “qvalue” package (version 2.18.0). Prognosis-related LR pairs associated with patient prognosis were determined as follows. 1) Storey’s q value <0.1 and 2) and HR > 1 (or HR < 1) in all cohorts.

### Consensus Clustering

Consensus clustering was used to classify the samples based on the gene expression data by constructing a consistency matrix using the R package “ConsensusClusterPlus” (Wilkerson and Hayes, 2010). The molecular subtypes of the samples were obtained using the significantly correlated LR pairs after screening. Here, we utilized the “pam” algorithm and “Canberra” as metric distances and performed 500 bootstraps, with each bootstrapping process including 80% of the patients in the training set. The number of clusters was set from 2 to 10, and the best classification was determined by calculating the consistency matrix and the consensus cumulative distribution function (CDF) (Senbabaoglu et al., 2014).

### Functional Annotations and Immune Infiltration Analysis

To investigate different gene expression patterns in each molecular subtype, we performed Gene Set Enrichment Analysis (GSEA v4.0) (Subramanian et al., 2005) using all candidate gene sets from the Hallmark database (Liberzon et al., 2015). Specifically, all samples of gene expression data were pooled by a pairwise grouping of patients and subjected to GSEA, and normalized *p*-value < 0.01 and FDR <0.05 were selected as the threshold for significance. Additionally, The “clusterProfiler” (Yu et al., 2012) package was used for functional annotation in up- or down-regulated genes for Kyoto Encyclopedia of Genes and Genomes (KEGG) analyses. In terms of the immune infiltration analysis, we used the deconvolutional methods of Cell-type identification by estimating relative subsets of RNA transcripts (CIBERSORT) (Newman et al., 2015) to estimate the fractions of the 22 immune cell subtypes. Additionally, to evaluate the immune and stromal cell abundance, the Estimation of STromal and Immune cells in Malignant Tumor tissues using the Expression data (ESTIMATE) (Becht et al., 2016) was performed. We used the default parameters of the TIDE program to assess the exclusion and dysfunction scores of tumor T cells (Jiang et al., 2018). The relative enrichment level of each KEGG pathway was represented by a ssGSEA score calculated from the R package GSVA (Hanzelmann et al., 2013).

### Genomic Data Analysis

The Simple Nucleotide Variation (SNV) dataset of the level4 of the TCGA samples processed by “MuTect2” was downloaded from GDC (https://portal.gdc.cancer.gov/). The SNP and CNV data were then analyzed and visualized using the “oncplot” function with the R package “maftools” (Mayakonda et al., 2018).

### Construction of the Ligand-Receptor score Model

The prognostic significant LR pairs were subjected to the penalized Cox model with L1-penalized Least Absolute Shrinkage and Selection Operator (LASSO) regression for computing personalized regression using the R package “glmnet” (Friedman et al., 2010). The model was validated with ten-fold cross-validations, and the corresponding log (λ) was selected based on the minimum partial likelihood of deviance, suggesting the most accurate model. According to the best log (λ), we got the number of predictors and their coefficients ( $\beta_{1}$ , $\beta_{2}$ , …, $\beta_{p}$ ). Then we utilized the Akaike information criterion (AIC), which took into account the statistical fit of the model and the number of parameters used to fit it. The “stepAIC” method in the “MASS” R package started with the most complex model and sequentially removed a variable to reduce the AIC. The smaller the value, the better the model, indicating that the model obtains an adequate fit with fewer parameters. The risk score of each patient was generated for the risk model.

LR.score = $\left(\beta_{1}x_{i1}+\;\beta_{2}x_{i2}+...+\;\beta_{p}x_{ip}\right)$ = 0.137∗ANXA1- > EGFR+0.212*CALR- > LRP1-0.358∗CCL14- > CCR5-0.118∗CHAD- > ITGA2-0.095∗DSPP- > ITGB6+0.071∗EFEMP1- > EGFR-0.253∗IFNE- > IFNAR2-0.367∗IL22- > IL22RA2+0.162∗PDGFD- > PDGFRB-0.115∗TNFSF13- > TNFRSF14.

### Drug Sensitivity Analysis

We downloaded drug sensitivity data for approximately 1000 cancer cell lines from Genomics of Drug Sensitivity in Cancer (GDSC) (http://www.cancerrxgene.org). Using the area under the curve (AUC) of antitumor drugs in cancer cell lines as a drug response indicator, we used Spearman correlation analysis to calculate the correlation between drug sensitivity and LR. score and considered |ρ |> 0.2, adjusted for FDR using Benjamini and Hochberg <0.05 were considered as a significant correlation. We also used the R package “pRRophetic” (Geeleher et al., 2014) for drug response prediction. The “IMvigor210CoreBiologies” R package was used to obtain 348 transcriptomes and corresponding clinical data from the IMvigor210 cohort of metastatic bladder cancer patients treated with an anti-PD-L1 drug (Atezolizumab) (Mariathasan et al., 2018).

### Protein Validations

We obtained the TMA (Tissue Microarrays) data from The Human Protein Atlas (HPA) database (https://www.proteinatlas.org). Candidate genes were projected into HPA and compared the staining intensity between low-grade and high-grade urothelial cancer. For genes with multiple antibodies used, we gave preference to antibodies with HPA as a prefix, as well as corresponding clinical information and staining intensity. The information of antibodies of each candidate genes were illustrated as below: CALR (HPA002242), LRP1 (HPA004182, HPA022903), PDGFD (HPA066271), PDGFRB (CAB003842), ANXA1 (HPA011271, HPA011272), EGFR (HPA001200, HPA018530), and EFEMP1 (HPA070841). Additionally, we validated the overall survival of the candidate genes in the protein level, based on the Cancer Proteome Atlas (TCPA) database (https://tcpaportal.org/tcpa/survival_analysis.html).

### Statistics

The unpaired Student’s t-test was used to analyze comparisons between two continuous variables and normally distributed variables. Non-normally distributed variables were analyzed with the Wilcoxon-rank sum test. To compare three or more groups, Kruskal-Wallis tests were performed for non-parametric methods. Fisher’s exact test was used to determine the associations between categorical variables. The correlation of the data was assessed using the Spearman rank correlation test. All the plotting and statistical analyses were performed using R version 4.1.3 (R Foundation for Statistical Computing, Vienna, Austria).

## Results

### Screening of Ligand-Receptor Pairs Associated With Patient Prognosis

To select the LR pairs associated with prognosis in bladder cancer patients, we firstly performed a survival analysis of LR pairs for each of these three cohorts (TCGA-BLCA, GSE13507, GSE32894) involved in our study. Then, based on the meta-analysis where we combined the prognostic significance *p*-values of LR pairs in the three bladder cancer cohorts and then corrected them by multiple testing. As a result, a total of 94 prognostic significant LR pairs were obtained, which contained 76 poor-prognosis LR pairs and 18 good-prognosis LR pairs (Figure 1B; Supplementary Table S2). The ligand and receptor networks are shown in Figure 1C. In terms of the functional annotations, we noticed there were several oncogenic pathways enriched among the significant LR pairs, including PI3K-AKT (adjust *p* = 2.80E-23), JAK-STAT (adjust *p* = 6.52E-09), RAP1 (adjust *p* = 3.61E-08), RAS (adjust *p* = 1.45E-07), and MAPK signaling pathways (adjust *p* = 1.73E-07) (Figure 1D).

### Molecular Classification of Ligand-Receptor Pairs

**FIGURE 2:**
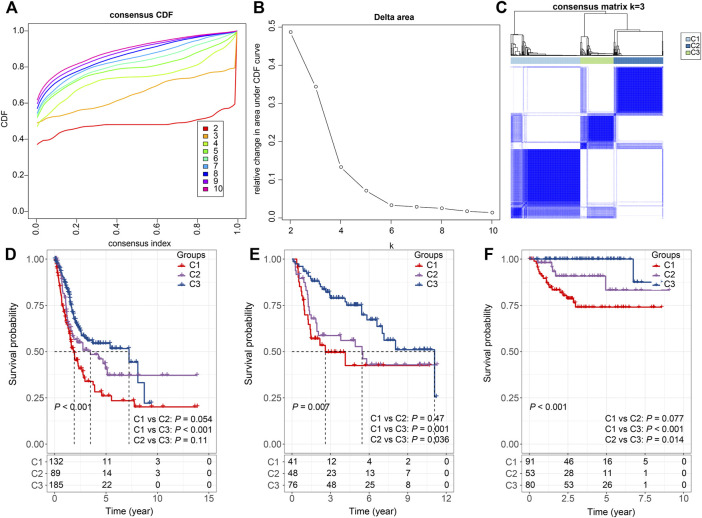
Consensus clustering based on LR pairs **(A)**. CDF curves of consensus clustering of the TCGA cohort **(B)**. Delta area curve of consensus clustering of the TCGA cohort. **(C)** Consensus matrices of identified clusters (k = 3) **(D)**. Kaplan–Meier curve of the overall survival across the three subtypes in the TCGA cohort (*p* < 0.001; C1 vs. C2: *p* = 0.054; C1 vs. C3: *p* < 0.001; C2 vs. C3: *p* = 0.11). **(E)**. Kaplan–Meier curve of the overall survival across the three subtypes in the GSE13507 cohort (*p* = 0.007; C1 vs. C2: *p* = 0.47; C1 vs. C3: *p* = 0.001; C2 vs. C3: *p* = 0.036). **(F)**. Kaplan–Meier curve of the overall survival across the three subtypes in the GSE32894 cohort (*p* = 0.007; C1 vs. C2: *p* = 0.077; C1 vs. C3: *p* < 0.001; C2 vs. C3: *p* = 0.014).

Furthermore, the sum of the receptor and ligand gene expressions was used as the expression intensity of 94 LR pairs, and these LR pairs were subjected to clustering. The 406 bladder cancer samples in the TCGA cohort were clustered into three subtypes (Figures 2A–C), with distinct prognosis (log-rank test, *p* < 0.001), where C1 was significantly associated with the worst patient outcome, and C3 exhibited the best survival probabilities (Figure 2D). To further validate the robustness and effectiveness of our classification system, we applied these 94 LR-pairs into two validation cohorts (GSE13507 and GSE32894). As expected, the consistency of the prognostic significance can be observed in the validation cohorts (log-rank test, *p* = 0.007 and *p* < 0.001, respectively) (Figures 2E,F).

**FIGURE 3:**
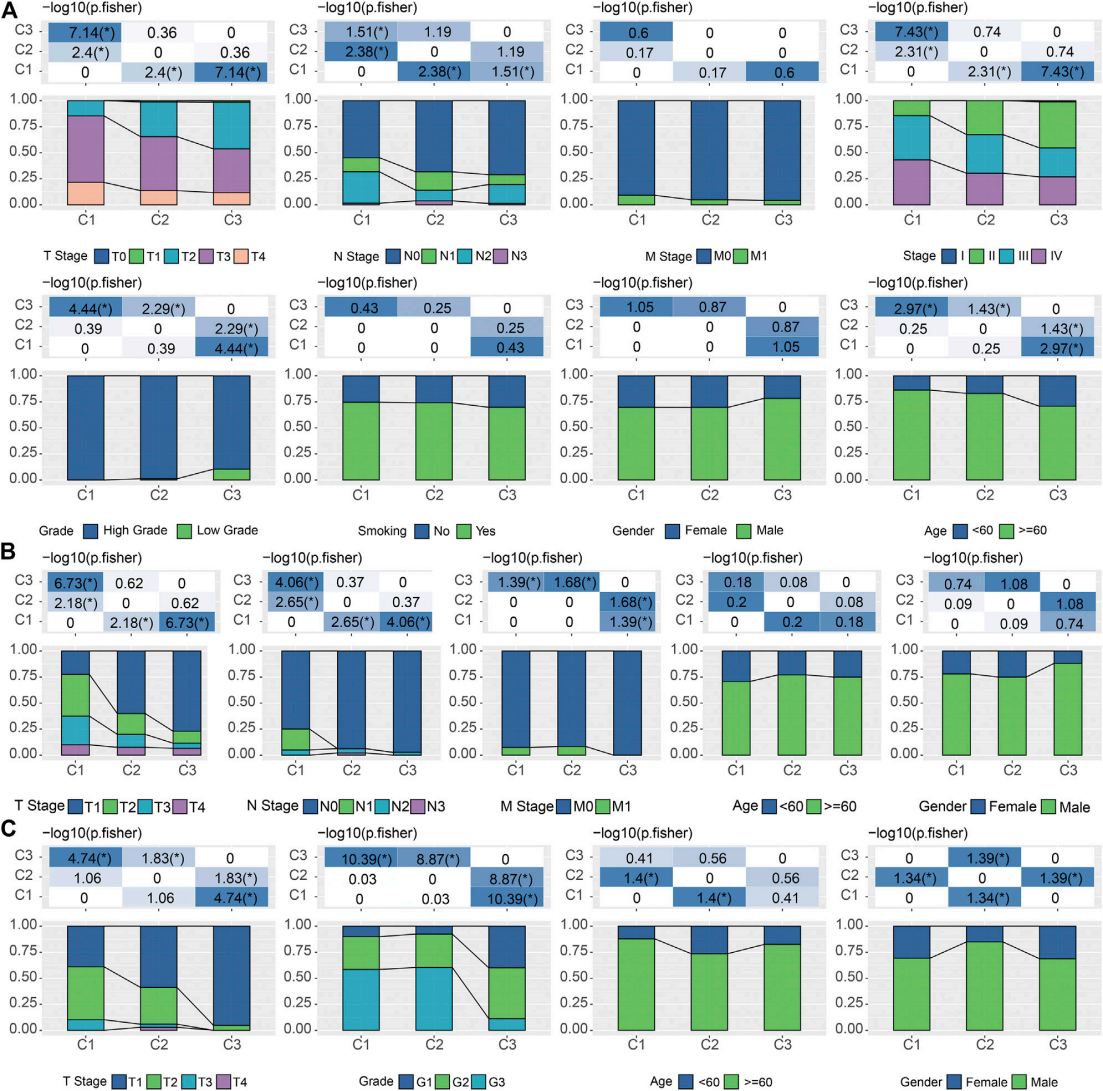
Correlations between LR pairs-based clusters and clinical characteristics **(A)**. The fraction of T stage, N stage, M stage, stage, grade, smoking, gender, and age, shown in C1, C2, and C3 groups in the TCGA cohort, followed by the corresponding comparison of the numbers in each group (Fisher’s exact test). *p < 0.05. **(B)**. The fraction of T stage, N stage, M stage, gender, and age, shown in C1, C2, and C3 groups in the GSE13507 cohort, followed by the corresponding comparison of the numbers in each group (Fisher’s exact test). *p < 0.05. **(C)** The fraction of T stage, N stage, M stage, gender, and age, shown in C1, C2, and C3 groups in the GSE32894 cohort, followed by the corresponding comparison of the numbers in each group (Fisher’s exact test). *p < 0.05.

Considering the substantial difference in prognosis among the three subtypes, we sought to investigate the clinical characteristics of the groups and determine the similarities and differences. In the TCGA cohort, there were no significant differences in M Stage, smoking, and gender among the three subtypes. In addition, we also noticed that C1 was significantly associated with high T stage, N stage, clinical stage, grade, and old age (Fisher’s exact test, −log10 *p* = 7.14, 1.51, 7.43, 4.44, and 2.97, respectively) (Figure 3A). And these phenomena could also be observed in the validation cohorts. C1 is significantly associated with a higher T stage in both GSE13507 (Fisher’s exact test, −log10 *p* = 6.73) and GSE32894 (Fisher’s exact test, −log10 *p* = 4.74) (Figures 3B,C).

### Functional Annotation of Ligand-Receptor Pairs-Based Clustering

**FIGURE 4:**
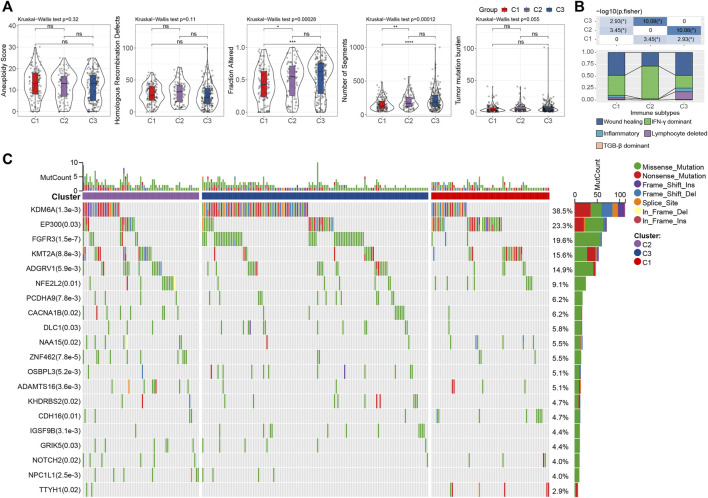
Genomic alternations of LR pairs-based clusters **(A)**. To differences of aneuploidy score, homologous recombination defects, number of segments, fraction altered, and tumor mutation burden in LR pairs-based clusters. ns, *p* > 0.05; *p < 0.05; **p < 0.01; ***p < 0.001. Kruskal-Wallis test. **(B)**. The fraction of bladder immune subtypes shown in C1, C2, and C3 groups in the TCGA cohort, followed by the corresponding comparison of the numbers in each group (Fisher’s exact test). *p < 0.05. **(C)** The mutational profiles of 404 Bladder cancer patients (top 20 frequently mutated genes). Top: mutation counts of the mutated genes in each patient. Bars: LR pairs-based clusters. Right: The types of genetic variants and their frequencies.

To investigate the genomic difference between the three subtypes, we obtained the genomic characteristics from the previous pan-cancer study (Thorsson et al., 2018). We were able to see that C1 has significantly lowest Fraction Altered and Number of Segments scores, but not Aneuploidy Score, Homologous Recombination Defects, and Tumor mutation burden (Wilcoxon rank-sum test, *p* < 0.05) (Figure 4A). Additionally, since five immune subtypes were identified in bladder cancer based on 160 immune signatures (Thorsson et al., 2018), we compared our LR pairs-based subtypes with the immune subtypes to test the effectiveness. Interestingly, C3 patients contained more inflammatory subtypes, which were reported as the best prognosis, high Th1:Th2, low proliferation, lowest intratumoral heterogeneity, as well as highest Th17 (Thorsson et al., 2018) (Figure 4B). Additionally, unlike C1 and C2, C3 has the lowest proliferation-dominated subtypes (e.g., Wound healing, IFN-γ dominant), somewhat reflecting the better patient outcomes in our study. Furthermore, we also compared the differences in gene mutations between different subtypes and showed the top 20 mutated genes (Figure 4C). As a result, we can see that the mutation frequencies of genes such as KDM6A and FGFR3 are significantly enriched in C3 (Figure 4C), where bladder cancers with FGFR3 mutations have been reported to target by the FDA-approved drug Erdafitinib (Loriot et al., 2019), and enzymatic inhibitor of EZH2 (Tazemetostat) has been suggested to target advanced urothelial carcinoma patients with KDM6A mutations (Meeks et al., 2020).

**FIGURE 5:**
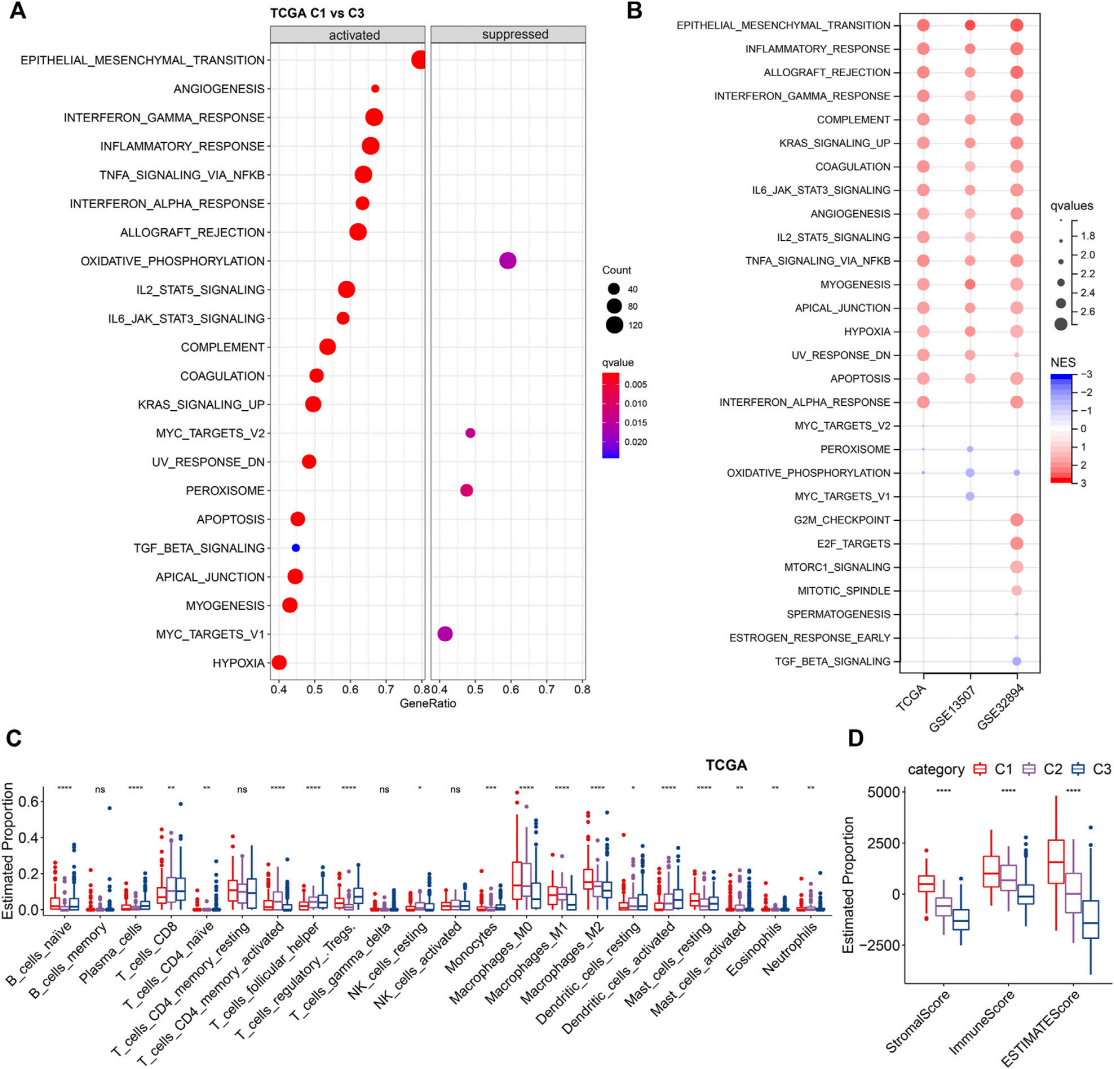
Enrichment analysis of LR-based clusters **(A)**. Dot plot of top 20 hallmarks of GSEA results between C1 and C3 in TCGA cohort. **(B)**. Dot plot of significant hallmarks of GSEA results in TCGA, GSE135607, and GSE32894 cohorts. Blue color indicates downregulated hallmarks; red color indicates upregulated hallmarks. **(C)**. The distribution of 22 immune cell types among LR-based clusters by CIBERSORT in TCGA cohort. ns, *p* > 0.05; *p < 0.05; **p < 0.01; ***p < 0.001. Kruskal-Wallis test. **(D)**. The distribution of stromal and immune scores among LR-based clusters by ESTIMATE in TCGA cohort. ns, *p* > 0.05; *p < 0.05; **p < 0.01; ***p < 0.001. Kruskal-Wallis test.

Next, we investigated the difference in hallmarks between different subtypes. By performing GSEA, a total of 17 hallmarks were significantly more activated in C1 than C3, including some immune-related gene sets (e.g., INFLAMMATORY RESPONSE, INTERFERON ALPHA RESPONSE, INTERFERON GAMMA RESPONSE, IL2_STAT5_SIGNALING, and IL6_JAK_STAT3_SIGNALING), suggesting high immune infiltrations of C1 (Figure 5A). These immune-related pathways could also be verified by the two validation cohorts, showing the same significance in C1. In addition, several other cancer-related pathways, including EPITHELIAL_MESENCHYMAL_TRANSITION, KRAS_SIGNALING_UP, TNFA_SIGNALING_VIA_NFKB, UV_RESPONSE_DN, etc., were significantly enriched in C1 (Figure 5B). Collectively, overall C1 patients showed an upregulated state in immune regulatory pathways, so we inferred that these ligand receptors used for molecular clustering might reflect the immune subtypes (wound healing, IFN-γ dominant) (Thorsson et al., 2018), i.e., C1 exhibits both hyperproliferative and infiltrative immune natures (Figure 4B). Using Cibersort deconvolutional approach, we found that C3 was characterized by the highest Tregs in both training and validation cohorts (Figure 5C, Supplementary Figures S1A,C). Additionally, by applying the ESTIMATE approach, we noticed that C1 had the highest stromal and immune scores (Figure 5D, Supplementary Figure S1B,D), which is consistent with the functional enrichment result that C1 represents a subtype with high immune infiltration.

### Construction of a Ligand-Receptor Pairs-Based Scoring Model

Since we found that the molecular subtypes based on LR pairs have different mutational landscapes, different pathway profiles, and different degrees of immune infiltration, we further compressed these 94 genes in the TCGA cohort using lasso regression to shrink the number of genes in the risk model, where we performed lasso cox regression. First, we analyzed the trajectory of each independent variable as shown in Supplementary Figure S2A, from which we can see that as the lambda gradually increased, the number of independent variable coefficients tending to zero also gradually increased. We used 10-fold cross-validation for model construction and analyzed the confidence interval under each lambda, as shown in Supplementary Figure S2B. We observed that the model reached optimality when lambda = 0.0375. We then chose 18 LR pairs at lambda = 0.0375 as the target LR pairs for the next step. Next, we performed a stepwise multi-factor regression analysis using the AIC criterion based on the 18 LR pairs from the lasso analysis results, which took into account the statistical fit of the model and the number of parameters used to fit. As a result, we identified 10 LR pairs as the key LR pairs, namely “ANXA1- > EGFR”, “CALR- > LRP1”, “CCL14- > CCR5”, “CHAD- > ITGA2”, “DSPP- > ITGB6”, “EFEMP1- > EGFR”, “IFNE- > IFNAR2”, “IL22- > IL22RA2”, “PDGFD- > PDGFRB”, “TNFSF13- > TNFRSF14”, as well as the results of multi-factor COX regression coefficients for these 10 LR pairs (Supplementary Figure S2C).

**FIGURE 6:**
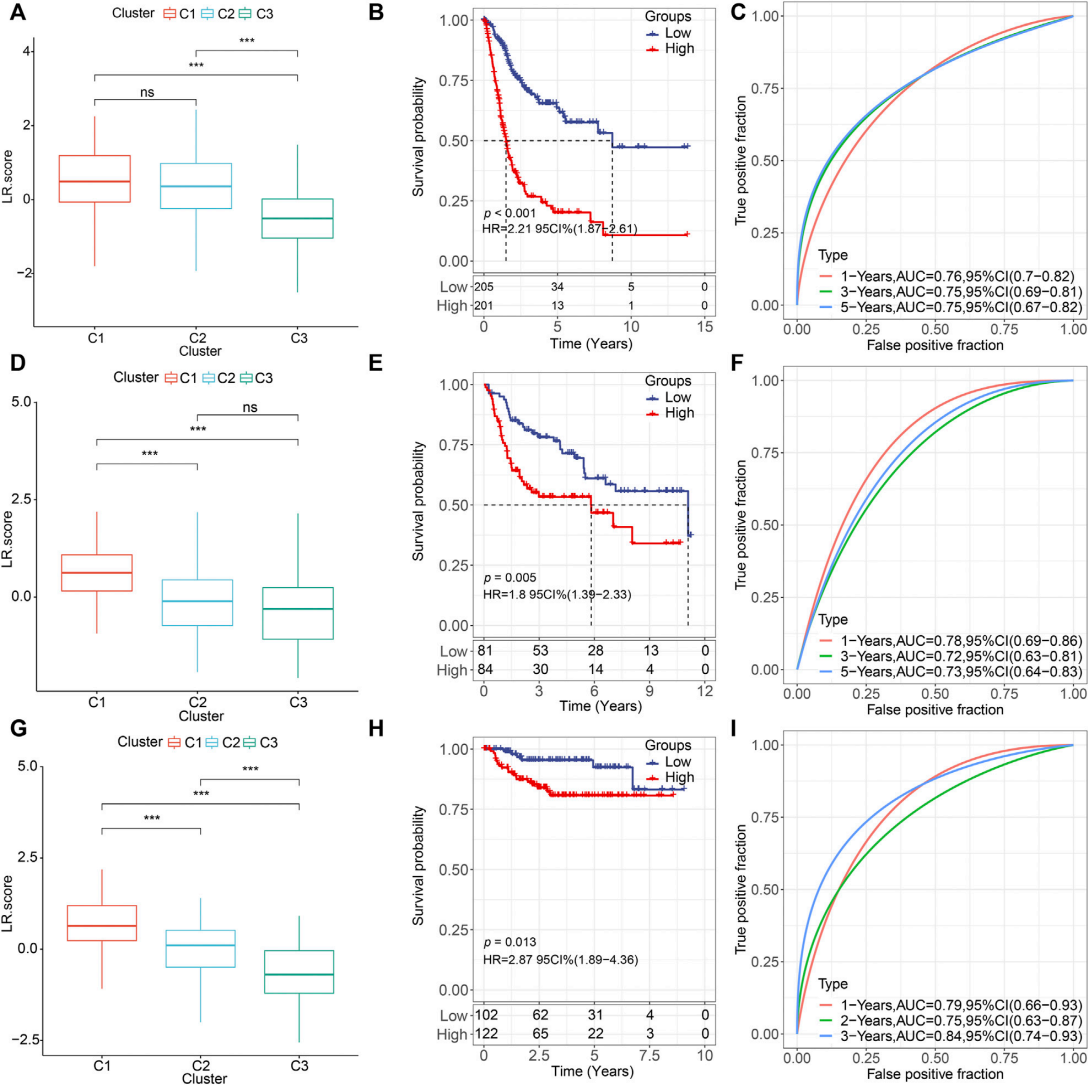
Prognostic effect of LR. score **(A,D,G)**. Distributions of the LR. score in the three LR-based clusters in **(A)** TCGA **(D)** GSE135607, and **(G)** GSE32894 cohorts. ns, *p* > 0.05; *p < 0.05; **p < 0.01; ***p < 0.001. Wilcoxon rank-sum test. **(B,E,H)**. Kaplan–Meier curve of the overall survival between low- and high-LR in **(B)** TCGA, **(E)** GSE135607, and **(H)** GSE32894 cohorts. **(C)**. ROC curve of the prognostic values of LR. score in TCGA cohort in 1-, 3-, and 5-year OS with AUC = 0.76, 0.75, and 0.75, respectively. **(F)**. ROC curve of the prognostic values of LR. score in TCGA cohort in 1-, 3-, and 5-year OS with AUC = 0.78, 0.72, and 0.73, respectively. **(I)**. ROC curve of the prognostic values of LR. score in TCGA cohort in 1-, 3-, and 5-year OS with AUC = 0.79, 0.75, and 0.84, respectively.

Next, we constructed an LR-pairs scoring model based on these 10 LR pairs to quantitatively analyze the LR-pairs pattern in bladder cancer patients, named LR. score. We found that the LR. score for subtype “C3” was significantly lower than that for “C1” and “C2” (Wilcoxon rank-sum test, *p* < 0.001) (Figure 6A). In order to further assess the clinical relevance of LR. score, we divided the patients into two groups with low and high LR. scores and determined its threshold score based on “0". Patients with low LR. score in the TCGA cohort showed a significant survival benefit (Figure 6B; log-rank test, *p* < 0.001). The AUC of the time-dependent ROC curve for LR. score was 0.76, 0.75, and 0.75 at 1, 3, and 5 years of overall survival, respectively (Figure 6C). We observed the same phenomena in the validation cohorts that in both GSE13507 and GSE32894, LR. score for subtype “C1” was significantly highest than that for “C2” and “C3” (Wilcoxon rank-sum test, *p* < 0.001) (Figures 6D,G). Patients with low LR. score in both cohorts also showed a significant survival benefit (Figures 6E,H; log-rank test, *p* = 0.005 and 0.013, respectively). In terms of the AUC of the ROC curves, the AUC values were all greater than 70% (1-, 3-, and 5-year OS predictions were 0.78, 0.72, and 0.73, respectively, in GSE13507; 1-, 3-, and 5-year OS predictions were 0.79, 0.75, and 0.84, respectively, in GSE32894), suggesting constant prognostic, predictive effects (Figures 6F,I).

### Clinical Characteristics of the Ligand-Receptor score

**FIGURE 7:**
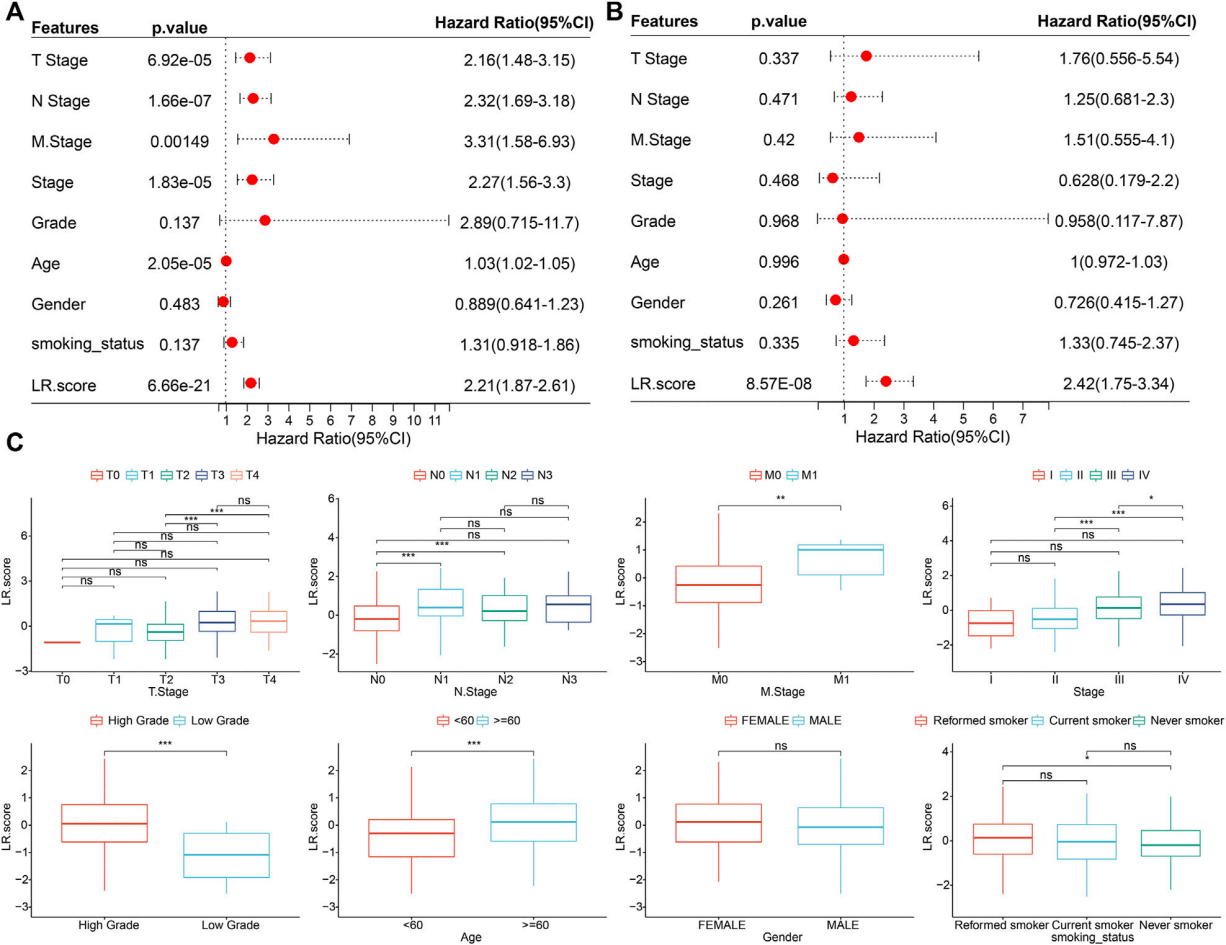
Associations between LR. score and clinical variables **(A)**. Univariate Cox regression analysis of overall survival with clinical factors of LR. score, patient age, gender, stage_T, stage_N, stage_M, stage, grade, smoking status in the TCGA cohort. **(B)**. Multivariate Cox regression analysis of overall survival with clinical factors of LR. score, patient age, gender, stage_T, stage_N, stage_M, stage, grade, smoking status in the TCGA cohort. **(C)**. Distributions of the LR. score in T stage, N stage, M stage, stage, grade, age, gender, and smoking status in TCGA cohort. ns, *p* > 0.05; *p < 0.05; **p < 0.01; ***p < 0.001. Wilcoxon rank-sum test.

To test whether the LR. score could be used as an independent prognostic factor, we performed univariate and multivariate Cox regression analyses using patients’ clinical characteristics (including age, gender, TNM, Stage, Grade, etc.). We found LR. score to be a reliable and independent prognostic biomarker for assessing patient prognosis (Figures 7A,B; HR = 2.42, 95% confidence interval 1.75–3.34, *p* = 8.57E-08). Next, we examined the relationship between LR. score and clinical characteristics. The results showed that the LR. score increased as the clinical grade and age increased. In brief, patients with higher clinical grades and older age had higher LR. scores (Figure 7C). In addition, as shown in Supplementary Figures S3A,B, we also compared the LR. score of patients in the GSE13507 and GSE32894 cohorts with clinicopathological characteristics and found that older and high-grade patients had higher LR. score scores.

### Ligand-Receptor score and Relevant Biological Functions

To investigate the relationship between LR. score and biological functions, we selected the gene expression profiles corresponding to bladder cancer samples in the TCGA cohort and performed ssGSEA to calculate the ssGSEA scores for each function corresponding to each sample (Supplementary Table S3). The correlation of pathways greater than 0.3 was selected as shown in Supplementary Figure S4, from which it can be seen that three pathways showed negative correlations, and 28 of the pathways showed positive correlations with the LR. score of the samples, including cancer-related pathways such as PATHWAYS_IN_CANCER, TGF_BETA_SIGNALING_PATHWAY, and WNT_SIGNALING_PATH WAY. Additionally, we explored the distributions of 22 immune cell abundance between high- and low- LR. scores. Most of the immune cells in the low LR. score group had significantly higher proportions than those in the high LR. score group, such as T_cells_CD8 (Wilcoxon rank-sum test, *p* < 0.001), T_cells_ follicular_helper (Wilcoxon rank-sum test, *p* < 0.01), T_cells_regulatory_Tregs (Wilcoxon rank-sum test, *p* < 0.001) (Supplementary Figure S5A). However, there was no significant difference in immune Scores between these two groups (Supplementary Figure S5B). Furthermore, we analyzed the relationship between LR. score and 22 immune cells. The scores of T_cells_CD8, T_cells_follicular_ helper, T_cells_regulatory_Tregs were significantly negatively correlated with LR. score while significantly positively correlated with Macrophages_M0 and Macrophages_M2 (Supplementary Figure S5C), indicating the different associations of adaptive and innate immunity between LR. scores.

**FIGURE 8:**
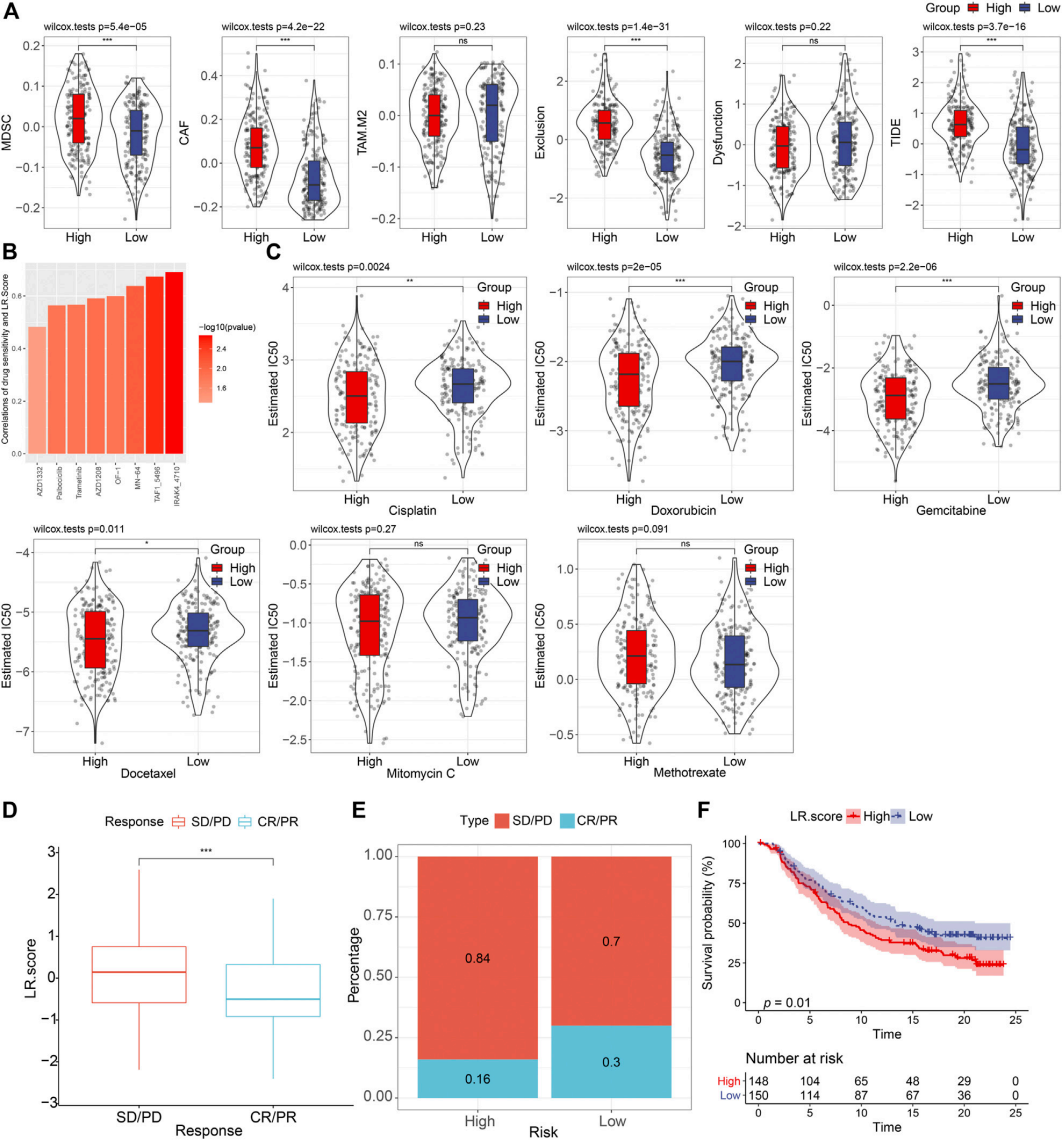
Drug predications based on the LR. score **(A)**. Distributions of MDSC, CAF, TAM. M2, T cell exclusion, T cell dysfunction, and TIDE score between high- and low-LR. score groups. ns, *p* > 0.05; ***p < 0.001. Wilcoxon rank-sum test. **(B)**. Bar plot of the spearman correlations of drug sensitivity and LR. Score. **(C)**. Distributions of the Estimated IC50 of Cisplatin, Doxorubicin, Gemcitabine, Docetaxel, Mitomycin C, and Methotrexate between high- and low- LR. score groups. ns, *p* > 0.05; *p < 0.05; **p < 0.01; ***p < 0.001. Wilcoxon rank-sum test. **(D)**. The fraction of SD/PD, and CR/PR, shown in high- and low-LR. score groups. **(F)**. Kaplan–Meier curve of the overall survival between high- and low- LR. score groups (*p* = 0.01).

Next, we used the TIDE software to assess the potential clinical effects of immunotherapy in our defined high and low LR. score groups (Supplementary Table S4). As shown in Figure 8A, we noticed that the higher TIDE score was found in, the lower LR. score group in the TCGA cohort (Wilcoxon rank-sum test, *p* = 3.7 e−16), suggesting that the high LR. score group had a higher likelihood of immune escape and a lower likelihood of benefiting from immunotherapy. We also compared the differences in predicted T-cell dysfunction scores and T-cell exclusion scores between high and low LR. score groups. The high LR. score group had the higher T-cell dysfunction scores, MDSC scores, and CAF scores (Wilcoxon rank-sum test, *p* = 1.4e−31, 5.4e−05, and 4.2e−22, respectively).

To further understand the impact of LR. score on drug response, we evaluated the relationship between LR. score and drug response in tumor cell lines. We identified eight significantly correlated pairs between LR. score and drug sensitivities in the Genomics of Drug Sensitivity in Cancer (GDSC) database, the highest correlation was represented in IRAK4_4710, followed by TAF1_5796, and MN-64, etc. (Spearman ρ > 0.6) (Figure 8B). We also assessed the chemotherapy responses between the high- and low- LR. score groups, where we analyzed several widely used drugs, including Cisplatin, Doxorubicin, Gemcitabine, Docetaxel, Mitomycin C, Methotrexate. Interestingly, patients with high LR. score scores were more sensitive to Cisplatin (Wilcoxon rank-sum test, *p* = 0.0024), Doxorubicin (Wilcoxon rank-sum test, *P* = 2e-05), Gemcitabine (Wilcoxon rank-sum test, *p* = 2.2e-06), and Docetaxel (Wilcoxon rank-sum test, *p* = 0.011) than the low LR. score group (Figure 8C).

To observe the relationship between LR. score and immunotherapy, we examined the ability of LR. score to predict patient response to ICB therapy. We found that in the anti-PD-L1 cohort (IMvigor210 cohort), 348 patients in the IMvigor210 cohort exhibited varying degrees of response to the anti-PD-L1 blockade, including complete response (CR), partial response (PR), stable disease (SD), and progressive disease (PD). Patients with SD/PD had a higher LR. score than patients with other types of response (Wilcoxon rank-sum test, *p* < 0.001) (Figure 8D). Low LR. score subgroups also showed significantly better treatment outcomes (Figure 8E) and significantly longer overall survival than the high LR. score subgroup (Figure 8F) (log-rank test, *p* = 0.01). Taken together, these results suggested that low LR. score group patients may benefit from anti-PD-L1 inhibitors.

### Independent Prognostic Factors Identification in the Risk Model

**FIGURE 9:**
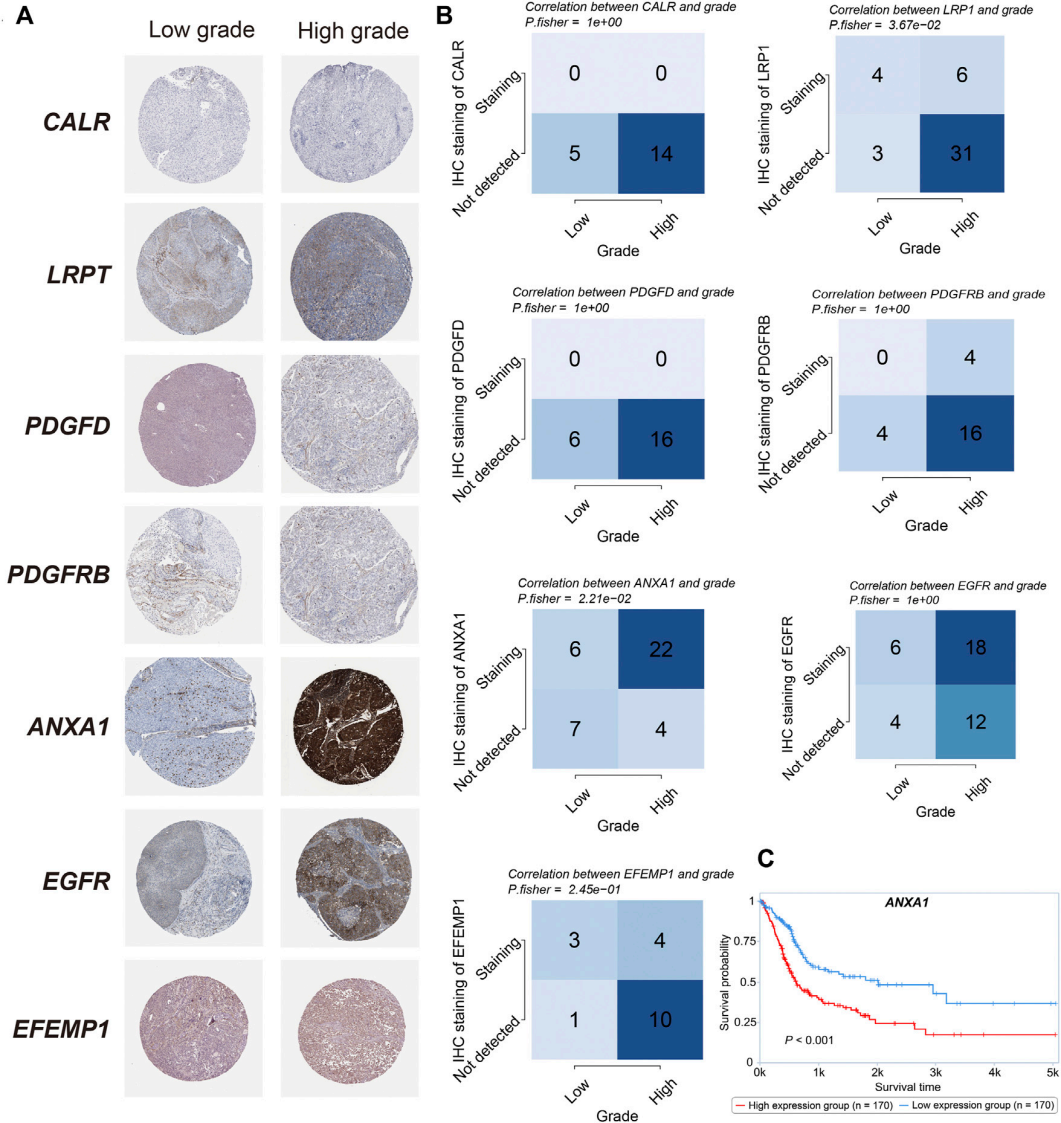
Protein validations **(A)**. IHC staining of CALR, LRP1, PDGFD, PDGFRB, ANXA1, EGFR, and EFEMP1 between high- and low-grade bladder cancer. **(B)**. The correlation between the grade and survival status and IHC staining in CALR, LRP1, PDGFD, PDGFRB, ANXA1, EGFR, and EFEMP1 (Fisher’s exact test). **(C)**. Kaplan–Meier curve of the overall survival between high- and low- ANXA1 expression in the protein level (*p* < 0.001).

Given that in biological and therapeutic systems upregulated genes are more likely to be targeted and regulated than downregulated genes, we decided to focus our validation study on investigating the protein levels of CALR, LRP1, PDGFD, PDGFRB, ANXA1, EGFR, and EFEMP1 involved in our risk model. As shown in Figures 9A,B, only ANXA1 significantly showed overexpression in high grade bladder cancer (*p* = 0.021, fisher-exact text). We also noticed that high expression of ANXA1 was significantly associated with overall survival in the protein level (log-rank test, *p* < 0.001) (Figure 9C). Taken together, we conclude that ANXA1 could be considered as an independent prognostic factor, and ANXA1- > EGFR may play critical roles in the tumorigenesis in bladder cancer.

## Discussion

In this study, we identified 94 prognostic LR pairs, which are mainly involved in JAK-STAT, PI3K-AKT, RAS, and MAPK signaling, suggesting certain LP pairs play a critical role in MIBC survival through the regulation of common oncogenic signaling pathways. We clustered three subtypes with distinct molecular features based on LP pairs associated with survival. We further constructed a 10 LP-pairs-based prognosis scoring model and validated the prediction power of this prognostic model in independent cohorts. Additionally, we found that this scoring model is able to predict chemotherapy response and immune checkpoint blockade treatment in bladder cancer. These results demonstrated that LP pairs-derived gene signatures could be potential biomarkers of prognosis and treatment response for MIBC.

The C1 subtype was featured by the hyperproliferative trait and worst prognosis. Upregulation of the functional hallmarks, including epithelial-mesenchymal transition, angiogenesis, and KRAS signaling, indicated that this subtype is enriched with the basal-like feature. Accordingly, chemotherapy may be considered for the C1 subtype. Consistently, higher LR. score in the C1 subtype predicted higher sensitivity to cisplatin, gemcitabine, and doxorubicin, which are the main first- and second-line chemotherapies in patients with MIBC. Additionally, the C1 subtype showed a higher immune score and stromal score. This subtype is in line with the consensus molecular classification, in which basal/squamous and stromal-rich types showed abundant immune cell infiltration (Kamoun et al., 2020). While the abundant immune cell infiltration suggests more sensitivity to anti-PD1/PD-L1 immunotherapies, the TIDE algorithm showed the C1 subtype might be less sensitive to ICB. The activation of interferon-gamma, inflammatory response, combined with TGF-beta signaling (a signaling pathway linked with immune escape in the tumor microenvironment), may contribute to complicating immune response in this subtype.

The high frequency of FGFR3 mutations (32%) in the C3 type suggested the shared characteristics with the luminal-papillary type according to the consensus classification (Kamoun et al., 2020). Correspondingly, the C3 subtype was associated with less advanced stage, low grade, and the best prognosis among the three subtypes. Targeting FGFR3 mutations with erdafitinib has been approved for advanced bladder cancer with an overall response of 40% (Loriot et al., 2019). The C3 subtype has a lower LR. score compared with the C1 subtype, which might be more sensitive to immune checkpoint immunotherapy. To date, erdafitinib, in combination with cetrelimab, an anti-PD-1 monoclonal antibody, has reported encouraging results in a phase 1b/2 NORSE (NCT03473743) study with an overall response of 68% and disease control rate of 90% (Powles T. B, 2021). In concordance with the preliminary results of this early-phase study, our findings suggested the synergic therapeutic potential of anti-FGFR3 targeted drug combined with anti-PD-1 immunotherapy.

The role of KDM6A as a tumor suppressor in bladder cancer has been studied both *in vitro* and *in vivo* (Nickerson et al., 2014). Additionally, KDM6A mutation is associated with a favorable prognosis. Our study also found the C3 type with the favorable prognosis has the most frequent KDM6A mutations (47%) among the three subtypes. Bladder cancer with KDM6A mutation increased susceptibility of EZH2 inhibitor through activation of natural killer cell signaling (Ramakrishnan et al., 2019). Tazemetostat, an EZH2 inhibitor, has been investigated to enhance the immune response of pembrolizumab (an anti-PD-1 monoclonal antibody) in the phase I/II trial (NCT03854474). As we identified the C3 subtype with potential response to both anti-PD-1 monoclonal antibody and EZH2 inhibitor, further studies are needed to test the combined treatment in this population.

We found that ligand-receptor pair of ANXA1−EGFR is associated with high grade and poor survival validated at protein expression level. Recently, showed that ANXA1 promotes bladder cancer progression *via* EGFR signaling Li et al. (2022). Silencing of ANXA1 inhibits bladder tumor growth using *in vitro* and *in vivo* models. These findings suggest that targeting ANXA1 could confer therapeutic benefit for treatment of bladder cancer.

Lower TIDE score was thought to predict anti-PD-1 immune response according to the TIDE algorithm (Jiang et al., 2018). The low LR. score group was with significantly lower TIDE score, predicting more response of PD-1 blockade (Figure 8A). While immune score did not significantly differ between the two LR. score groups, higher abundance of CD8 T cells and Treg cells in the low LR. score group may partly explain this prediction. Abundance of intratumor PD-1 Treg was known to suppress anti-tumor immunity (Palucka and Coussens, 2016). In addition, high abundance of CD8+T cells at exhausted state, which was featured with high their high PD-1 expression, lost the proper effector response to eliminate tumor cells (Budimir et al., 2022). Taken together, PD-1/PD-L1 blockade could confer better immune response in the low LR. score group through inhibiting PD-1 expression Treg cells and reactivating the exhausted CD8 T cells, which may further restore the cytotoxic effect in the tumor microenvironment.

In our study, the LR. score demonstrated significant survival prediction in an independent bladder cancer cohort, suggesting the ligand-receptor pair network is critically involved in the prognosis of bladder cancer. Moreover, Lower LR. score was associated with a better response to anti-PD-L1 treatment, which was validated in the IMvigor 210 cohort. Conversely, Higher LR. score was associated with a higher chemotherapy response. These findings suggested that the susceptible populations to chemotherapy and immunotherapy might be different. Intriguingly, this finding is not in agreement with the main results of two phase III studies, Keynote-361 and IMvigor130, which investigated the synergistic potential of pembrolizumab and atezolizumab adding to the standard chemotherapy for advanced bladder cancer, respectively (Galsky et al., 2020; Powles T et al., 2021). In these two studies, anti-PD-1/PD-L1 treatment in addition to platinum-based chemotherapy did not show a significantly greater response or progression-free survival compared with chemotherapy alone. While the mechanisms remain unclear, the overlapping features of the basal type sensitive to both chemotherapy and immunotherapy may in part explain the negative results of the two trials. Further studies to identify different responsive populations using the LR. score may provide a scientific basis for combined treatment with chemotherapy and ICB.

In conclusion, our study showed that the network of LR pairs in bladder cancer is associated with prognosis. We identified three LR-derived subtypes significantly associated with prognosis. These subtypes have distinct clinical and molecular characterizations. The development of LR. score using 10 LR pairs served as an independent prognostic tool and also could be used to guide treatment decisions, including chemotherapy or immunotherapy, which are the two main treatment modalities for advanced bladder cancer. We confirmed that ANXA1 is associated with higher grade and poor prognosis in bladder cancer, which provides a basis for future development of therapeutic agents.

## Data Availability

The dataset were from public TCGA and GEO datasets. The datasets presented in this study can be found in online repositories. The names of the repository/repositories and accession number(s) can be found in the article.

## Supplementary Material

Supplementary Figure S1
**(A)**. The distribution of 22 immune cell types among LR-based clusters by CIBERSORT in GSE13057 cohort. ns, *p* > 0.05; * *p* < 0.05; ** *p* < 0.01; ****p* < 0.001. Kruskal-Wallis test. **(B)**. The distribution of stromal and immune scores among LR-based clusters by ESTIMATE. ns, *p* > 0.05; * *p* < 0.05; ** *p* < 0.01; ****p* < 0.001. Kruskal-Wallis test in GSE13507 cohort. **(C)**. The distribution of 22 immune cell types among LR-based clusters by CIBERSORT in GSE32894 cohort. ns, *p* > 0.05; * *p* < 0.05; ** *p* < 0.01; ****p* < 0.001. Kruskal-Wallis test. **(D)**. The distribution of stromal and immune scores among LR-based clusters by ESTIMATE. ns, *p* > 0.05; * *p* < 0.05; ** *p* < 0.01; ****p* < 0.001. Kruskal-Wallis test in GSE32894 cohort.

Supplementary Figure S2
**(A)**. The trajectory of each LR pair, the horizontal axis represents the logarithmic value of each lambda, and the vertical axis represents the coefficient value. **(B)**. Partial likelihood deviance under each ln (lambda) of the LASSO Cox regression model. **(C)**. The Lasso Cox coefficients of significant risk and protective LR pairs.

Supplementary Figure S3
**(A)**. Distributions of the LR.score in T stage, N stage, M stage, stage, grade, age, and gender, in GSE13507 cohort. ns, *p* > 0.05; * *p* < 0.05; ** *p* < 0.01; ****p* < 0.001. Wilcoxon rank-sum test. **(B)**. Distributions of the LR.score in T stage, grade, age, and gender, in GSE32894 cohort. ns, *p* > 0.05; * *p* < 0.05; ** *p* < 0.01; ****p* < 0.001. Wilcoxon rank-sum test.

Supplementary Figure S4 The correlations between LR.score and KEGG pathway scores. In the upper right triangle, the gradient of color represents the level of correlation, whereas in the lower left triangle, the gradient of color represents−log10 (*p* value). **p* < 0.05; ***p* < 0.01, ****p* < 0.001.

Supplementary Figure S5
**(A)**. The distribution of 22 immune cell types among between high- and low- LR.score groups by CIBERSORT in TCGA cohort. ns, *p* > 0.05; * *p* < 0.05; ** *p* < 0.01; ****p* < 0.001. Kruskal-Wallis test. **(B)**. The distribution of stromal and immune scores between high- and low- LR.score groups by ESTIMATE in TCGA cohort. ns, *p* > 0.05; * *p* < 0.05; ** *p* < 0.01; ****p* < 0.001. Kruskal-Wallis test. **(C)**. The correlations between LR.score and immune cell GSVA scores. In the upper right triangle, the gradient of color represents the level of correlation, whereas in the lower left triangle, the gradient of color represents−log10 (*p* value). **p* < 0.05; ***p* < 0.01, ****p* < 0.001.

Supplementary Table S1 All Ligand Receptor (LR) pairs.

Supplementary Table S2 94 prognostic significant LR pairs.

Supplementary Table S3 ssGSEA scores of the KEGG pathways in TCGA cohort.

Supplementary Table S4 TIDE score of each sample in TCGA cohort.
