# Supplementary figures and images for: Characterization of Ligand-Receptor Pair in Bladder Cancer Develops a Validated Scoring Model for Prognosis and Treatment Response
Source: Front Cell Dev Biol. 2022 Jun 17;10:915798. doi: 10.3389/fcell.2022.915798 (PMC9247554; doi:10.3389/fcell.2022.915798)

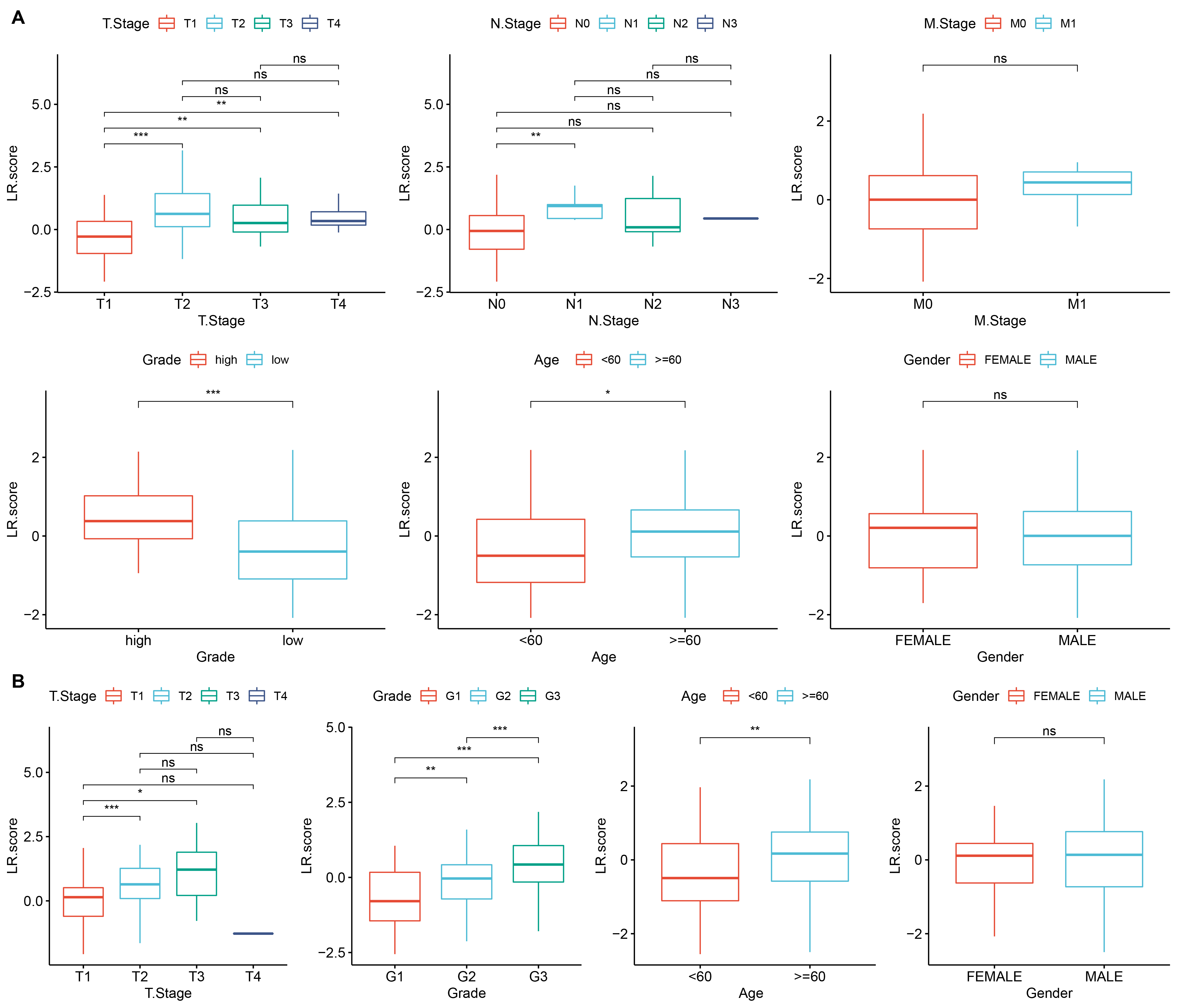

Supplement: Supplementary file 1 [file Image3.TIFF]

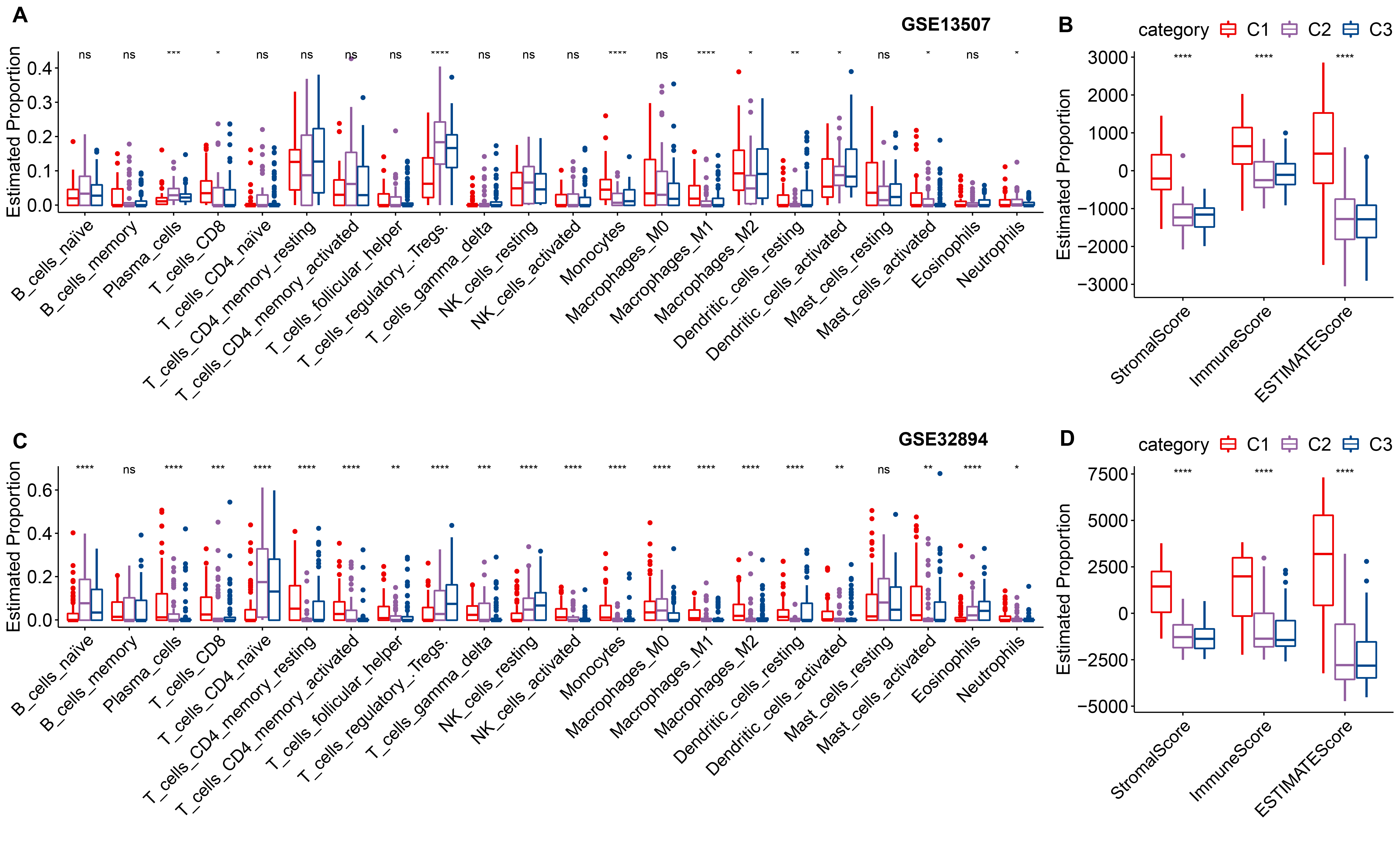

Supplement: Supplementary file 2 [file Image1.TIFF]

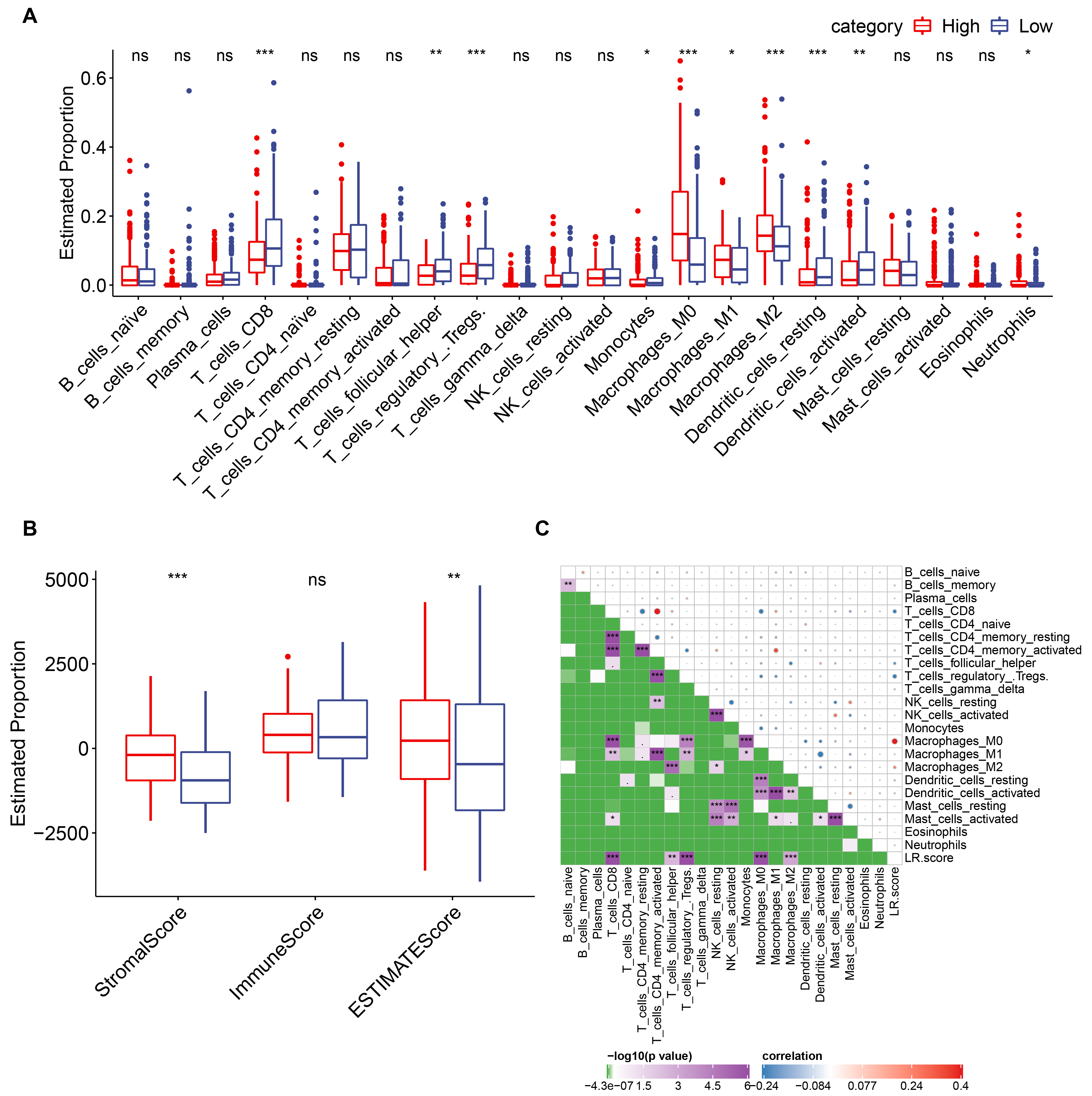

Supplement: Supplementary file 3 [file Image5.TIFF]

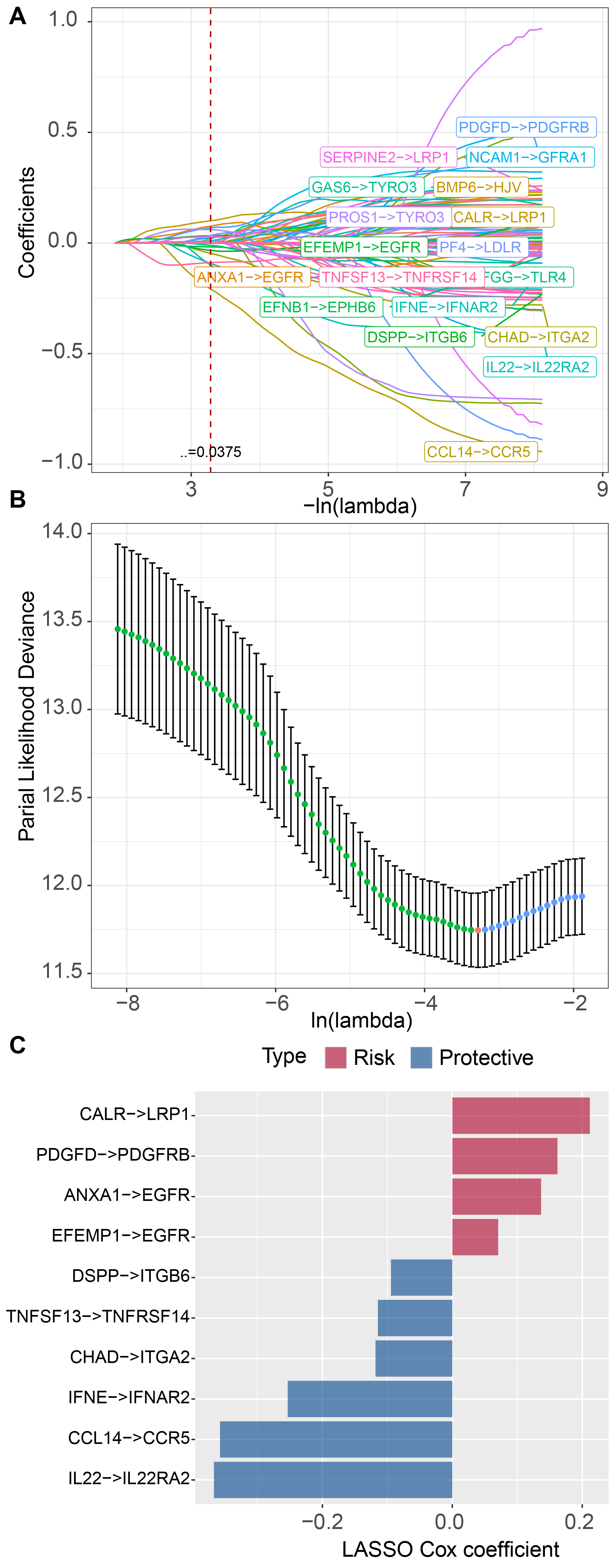

Supplement: Supplementary file 4 [file Image2.TIFF]

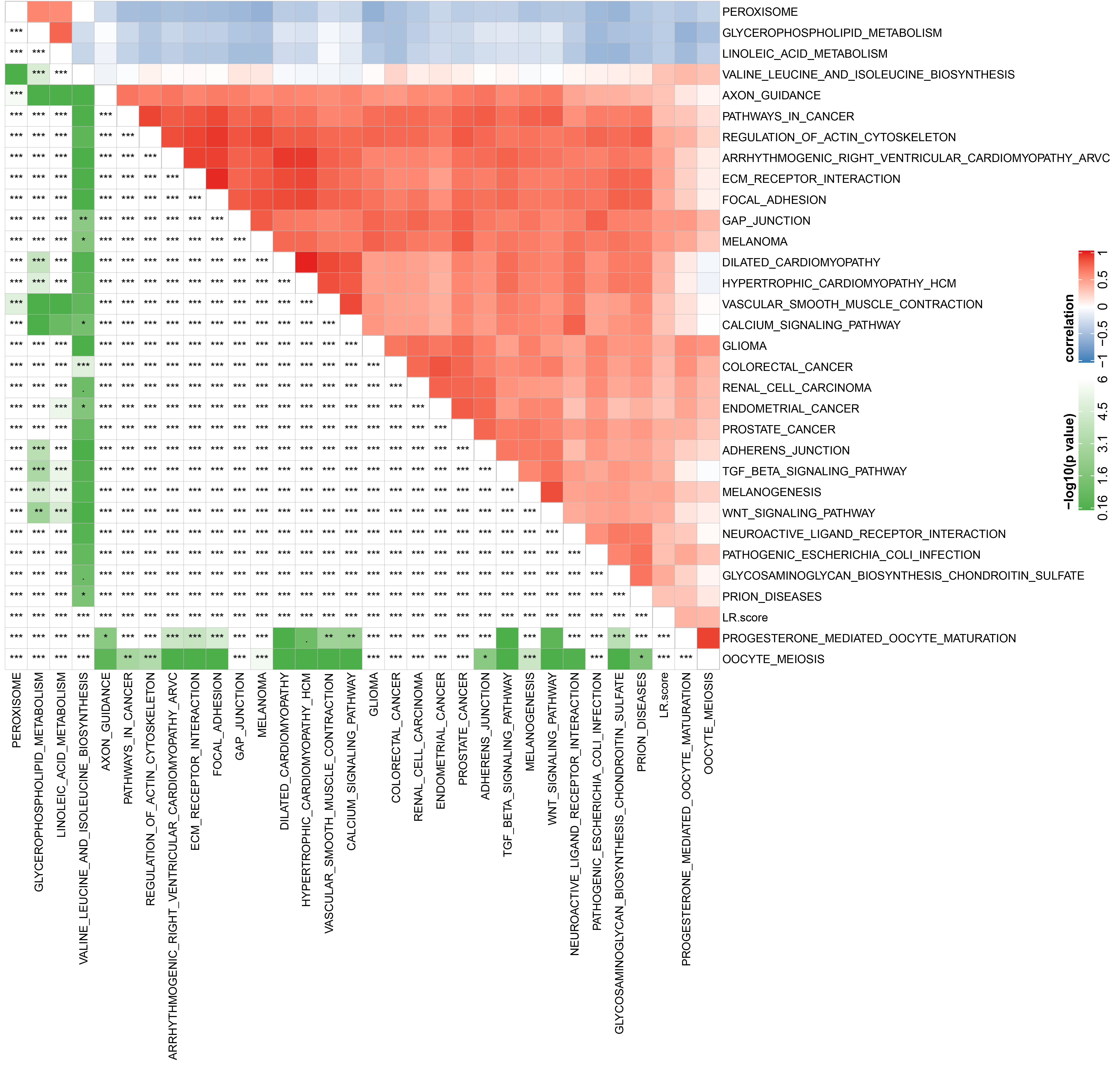

Supplement: Supplementary file 5 [file Image4.TIFF]
